# Mechanism of the improvement of the energy of host–guest explosives by incorporation of small guest molecules: HNO_3_ and H_2_O_2_ promoted C–N bond cleavage of the ring of ICM-102

**DOI:** 10.1038/s41598-021-89939-1

**Published:** 2021-05-18

**Authors:** Yiwen Xiao, Lang Chen, Kun Yang, Deshen Geng, Jianying Lu, Junying Wu

**Affiliations:** State Key Laboratory of Explosion Science and Technology, Beijing Institute of Technology, Beijing, 100081 China

**Keywords:** Reaction kinetics and dynamics, Atomic and molecular collision processes

## Abstract

Host–guest materials exhibit great potential applications as an insensitive high-energy–density explosive and low characteristic signal solid propellant. To investigate the mechanism of the improvement of the energy of host–guest explosives by guest molecules, ReaxFF-lg reactive molecular dynamics simulations were performed to calculate the thermal decomposition reactions of the host–guest explosives systems ICM-102/HNO_3_, ICM-102/H_2_O_2_, and pure ICM-102 under different constant high temperatures and different heating rates. Incorporation of guest molecules significantly increased the energy level of the host–guest system. However, the initial reaction path of the ICM-102 molecule was not changed by the guest molecules. The guest molecules did not initially participate in the host molecule reaction. After a period of time, the H_2_O_2_ and HNO_3_ guest molecules promoted cleavage of the C–N bond of the ICM-102 ring. Stronger oxidation and higher oxygen content resulted in the guest molecules more obviously accelerating destruction of the ICM-102 ring structure. The guest molecules accelerated the initial endothermic reaction of ICM-102, but they played a more important role in the intermediate exothermic reaction stage: incorporation of guest molecules (HNO_3_ and H_2_O_2_) greatly improved the heat release and exothermic reaction rate. Although the energies of the host–guest systems were clearly improved by incorporation of guest molecules, the guest molecules had little effect on the thermal stabilities of the systems.

## Introduction

Synthesis of new high-performance energetic materials is always a goal of researchers. In recent years, a series of new host–guest energetic materials have been obtained through the host–guest inclusion strategy, such as CL-20/H_2_O_2_^1^ and the HGI-1/2/3 series^2^, which are composed of an insensitive high-energy–density explosive ICM-102^3,4^. The usual way is to embed the guest oxidizing small molecules in the lattice voids of the high energy explosive. The relative position relationship between the voids and the host explosive molecules determines the ratio of host/guest molecules. Generally, a hydrogen bond will form between a H atom of the guest molecule and an O atom (or another atom) of the host explosive to maintain the stability of the host–guest structure.

There are significant differences in the energy densities, detonation performance, and other aspects of host–guest systems obtained by adding different guest molecules^5^. For instance, Xu et al.^6^ compared the calculated detonation velocity/pressure values of HNIW/N_2_O and HNIW/CO_2_, and the results all showed that the detonation velocity/pressure of HNIW/N_2_O was superior to that of HNIW/CO_2_. For guest molecules, the difference in the elemental composition leads to a different influence on the reaction mechanism of the host explosive. However, at present, it is difficult to obtain the mechanisms of different guest molecules participating in and influencing the reactions of the host explosives through macroscale experimental characterization methods. In addition, the mechanisms of the improvement of the energies of the host–guest explosives by guest molecules are also of interest.

In recent years, molecular dynamics (MD) simulations based on the ReaxFF^7^ reactive force field have provided a good choice to investigate the chemical reaction mechanisms of energetic materials under extreme conditions. Since Strachan et al. used ReaxFF to calculate the chemical reactions of RDX explosives under shock^8^ and high temperatures^9^, the ReaxFF MD method has been used to investigate explosive reactions. Furman et al.^10^ used the ReaxFF-lg^11^ reactive force field and added the long-range interactions between molecules to clarify the difference in the activation energies between gas phase and condensed phase TNT explosives from the reaction mechanism for the first time. Guo et al.^12^ used the ReaxFF-lg force field to explain why addition of TNT molecules results in a decrease in the sensitivity of the co-crystal explosive CL-20/TNT from the perspective of the microchemical reaction. Research on single-compound explosives^13–15^ and co-crystal explosives^16,17^ has been extensive; however, there are few studies related to the reaction mechanisms of host–guest explosives^18^ under extreme conditions. In addition, it is still unclear how different guest molecules affect the reaction mechanism and thermal reaction kinetic parameters of the host molecules.

In this study, the MD method based on ReaxFF-lg was used to calculate the thermal decomposition reactions of the host–guest explosive systems ICM-102/HNO_3_ and ICM-102/H_2_O_2_, and pure ICM-102 under different constant temperatures (2500, 2750, 3000, 3250, and 3500 K) and programmed heating (heating rates of 50 and 100 K ps^−1^). The mechanisms of the energy improvement of the host–guest explosives by the guest molecules are discussed in detail. The initial reaction paths between the host and guest molecules in the systems are clarified. The effect of the guest molecules on the kinetic parameters in the endothermic/exothermic reaction stage of thermal decomposition of the host–guest systems was investigated, and the effect of the guest molecules on formation of the main final small molecules products was analyzed.

## Computational methods

### Computational model

**Figure 1:**
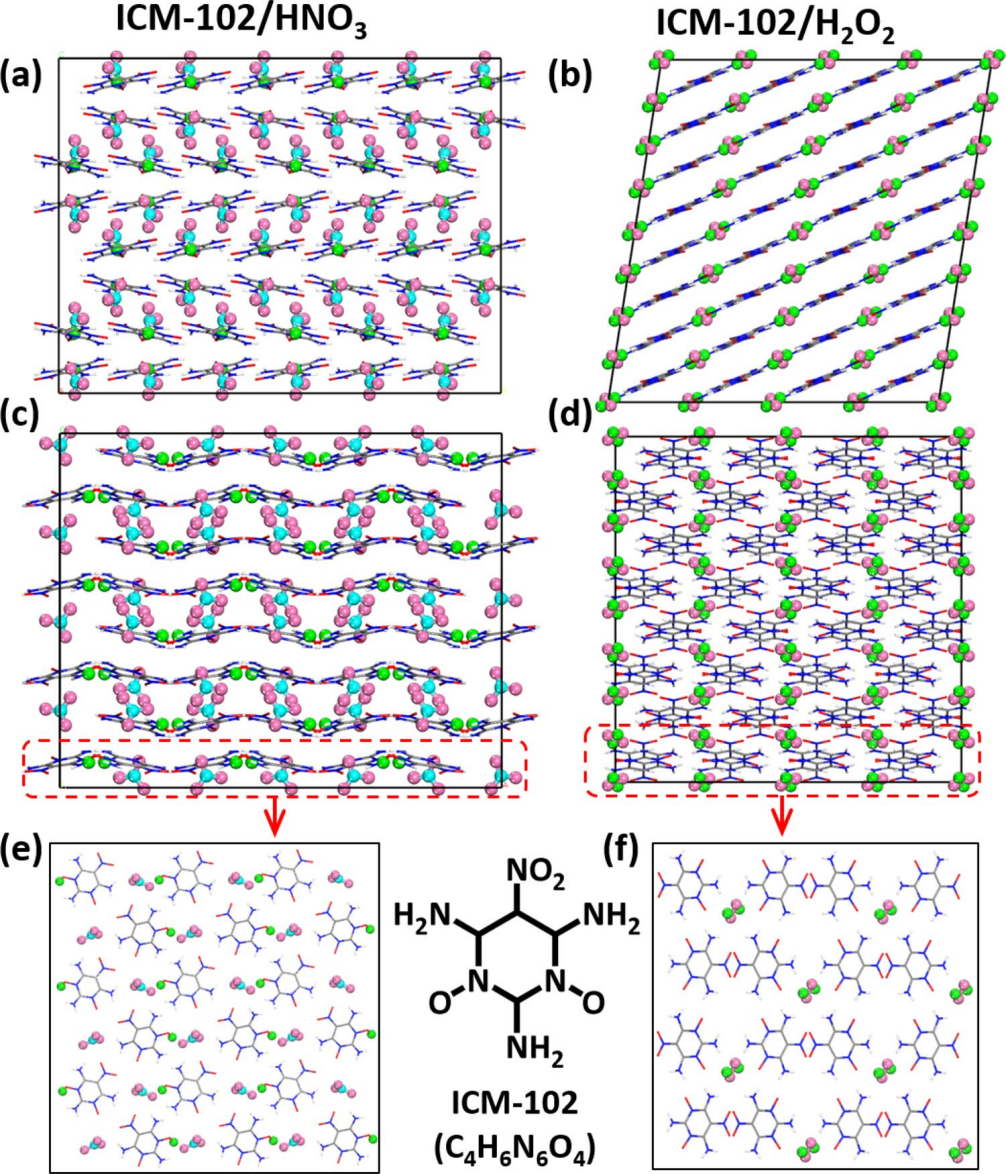
Initial unit cell structures. The (**a**) and (**b**) is a point of view from a-axis, (**c**) and (**d**) a point of view from b-axis, (**e**) and (**f**) is one layer of view from c-axis. The C, H, N, and O atoms of the host ICM-102 molecules are gray, white, blue, and red, respectively. The H, N, and O atoms of the guest molecules are green, cyan, and pink, respectively. The same representations will be used hereafter.

The initial ICM-102/H_2_O_2_ and ICM-102/HNO_3_ unit cell structures were obtained from the Cambridge Crystallographic Data Centre (CCDC, CCDC numbers 1831628 and 1887848). Both of the unit cells contain eight ICM-102 molecules, and the difference is the number of guest molecules: ICM-102/HNO_3_ contains eight HNO_3_ molecules and ICM-102/H_2_O_2_ contains four H_2_O_2_ molecules. We enlarged the unit cells along the *a*, *b*, and *c* axes to construct a 3 × 3 × 2 ICM-102/HNO_3_ supercell containing 3600 atoms and a 2 × 2 × 4 ICM-102/H_2_O_2_ supercell containing 2816 atoms (Fig. 1). In order to test whether the size of the system affects the calculation results, we performed some simulations with a larger supercell (Fig. S1). The law of energy release excellently agrees with the smaller system. The pure ICM-102 supercell for calculation comparison was obtained by removing the HNO_3_ molecules. Each of the three supercells was relaxed for sufficient time to guarantee that the molecules were in reasonable positions at relatively low energies before formal calculation.

### Simulation method

All of the simulations were performed with the LAMMPS MD simulator based on the ReaxFF-lg reactive force field. First, the equilibrium structures of the supercells at 0 K were obtained by energy minimization with the conjugate gradient algorithm. The initial velocities were assigned to the atoms according to the Maxwell–Boltzmann distribution at 298 K. The Berendsen thermostat was used to relax the supercells with the canonical ensemble (NVT) at 298 K for 10 ps. The equilibrium supercell structures were then obtained by an isobaric–isothermal (NPT) MD simulation at 0 Pa and 298 K for 10 ps. The structures of all of the molecules remained intact during the whole relaxation simulations. NVT MD simulations with the Berendsen thermostat were performed to heat the supercell systems to the target temperatures (2500, 2750, 3000, 3250, and 3500 K) until the potential energy (PE) stabilized or to heat the systems from 300 to 3000 K in 54 ps (heating rate ~ 50 K ps^−1^) or 27 ps (heating rate ~ 100 K ps^−1^). The time step was 0.1 fs, and periodic boundary conditions were applied. The atom trajectories, molecular species, and bonds between the atoms were recorded every 50 fs. We used a bond order ≥ 0.3 to judge formation of chemical bonds^19,20^.

It has been reported that the ReaxFF-lg force field can effectively describe the reactions of ICM-102^21,22^. To verify the applicability of the ReaxFF-lg force field for ICM-102/HNO_3_ and ICM-102/H_2_O_2_, we compared the cell parameters after relaxation at 298 K and 0 Pa with the initial parameters from the CCDC. A comparison of the lattice parameters and densities of ICM-102/HNO_3_ and ICM-102/H_2_O_2_ is given in Table 1. The lattice parameters after relaxation by the ReaxFF-lg were not considerably different from the initial parameters in the CCDC database, which shows that the ReaxFF-lg force field can describe ICM-102/HNO_3_ and ICM-102/H_2_O_2_ reactions.

**Table 1:** Comparison of the lattice parameters and densities of ICM-102/HNO_3_ and ICM-102/H_2_O_2_.

Crystal	Method	*a*/Å	*b*/Å	*c*/Å	*ρ*/g cm^−3^
ICM-102/HNO_3_	From CCDC	33.717	35.607	27.022	1.954
	ReaxFF-lg	34.051	35.960	27.290	1.902
ICM-102/H_2_O_2_	From CCDC	28.256	29.392	29.660	1.913
	ReaxFF-lg	28.267	29.404	29.672	1.911

### Calculation methods of the kinetic parameters in different reaction stages

The thermal decomposition reaction of explosives can be divided into three stages: the initial decomposition stage, intermediate decomposition stage, and final product evolution stage^23^. Through fitting the calculation results, the reaction rates/product formation rate constants and activation energies in the different stages were obtained to characterize the kinetic parameters of the thermal decomposition reaction at high temperatures.

The initial decomposition stage is an endothermic process, and it can also be called the endothermic reaction stage. The ICM-102 molecules decompose and absorb heat in this stage, so we used the change of the number of ICM-102 molecules to calculate the reaction rate in this stage. The change of the number of ICM-102 molecules with time was fitted by a first-order decay exponential function:

1 $$ N(t) = N_{0} \times \exp [ - k_{1} (t - t_{0} ) ] $$

where *N*_0_ is the initial number of ICM-102 molecules, *t*_0_ is the time when ICM-102 starts to decompose, and *k*_1_ is the initial decomposition stage rate constant.

The intermediate exothermic stage of the thermal decomposition reaction then occurs. In this stage, the molecules in the system react violently and rapidly release a large amount of heat, so the heat release rate of the system is used to express the reaction rate in this stage. The PE curves were fitted by a first-order decay exponential function:

2 $$ U(t) = U_{\infty } (T) + \Delta Q(T) \times \exp [ - k_{2} (t - t_{\max } ) ] $$

where *U*_∞_(*T*) is the asymptotic value of PE (kcal mol^−1^), Δ*Q*(*T*) is the system heat released (kcal mol^−1^), *k*_2_ is the intermediate decomposition stage rate constant, and *t*_max_ is the time of the maximum PE.

Finally, the changes of the numbers of main final products N_2_, CO_2_, and H_2_O at different temperatures were fitted by

3 $$ C(t) = C_{\infty } \{ 1 - \exp [ - k_{3} (t - t_{i} ) ]\} $$

where *C*_∞_ is the asymptotic number of the product, *k*_3_ is the formation rate constant of the product, and *t*_i_ is the time of appearance of the product.

## Results and discussion

### Improvement of the energy of host–guest systems by small guest molecules

**Figure 2:**
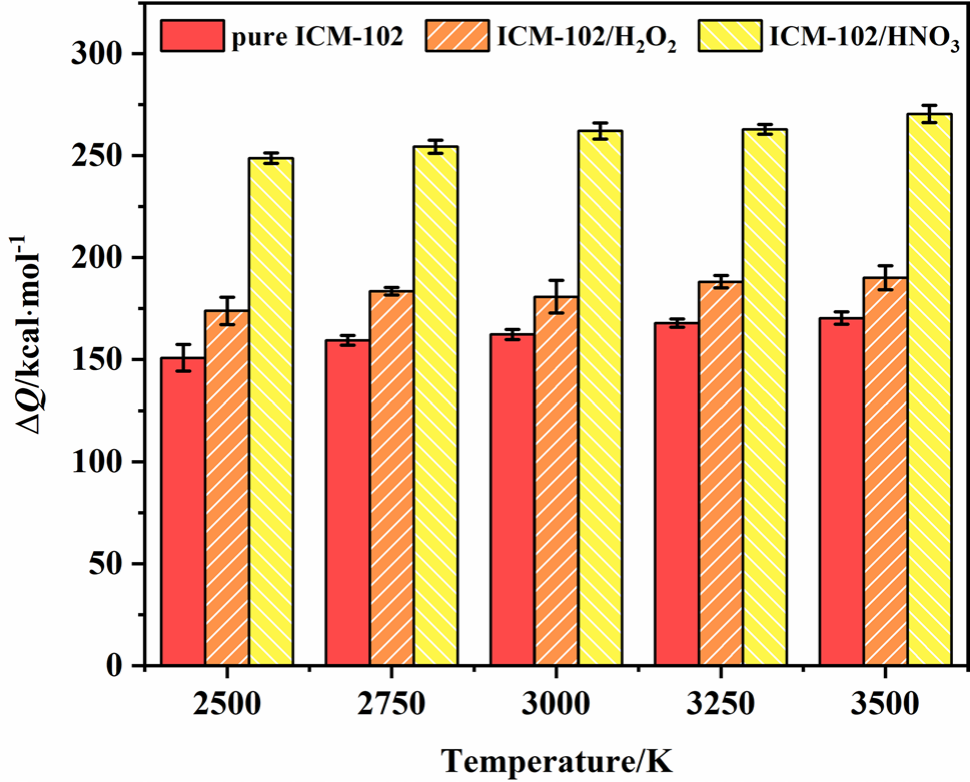
Comparison of the average heat release of each ICM-102 molecule in the pure ICM-102, ICM-102/H_2_O_2_, and ICM-102/HNO_3_ systems at different temperatures.

**Figure 3:**
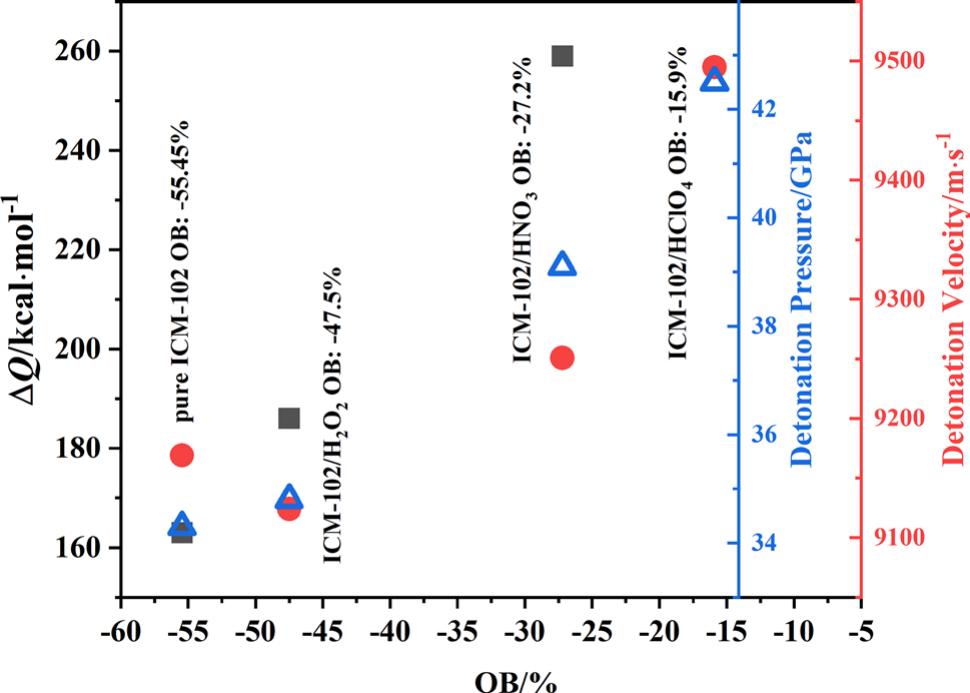
Comparison of the heat release (at 3000 K) and detonation pressure/velocity values of the different host–guest systems.

The improvement of the energy level of the system is reflected in the heat release, detonation pressure/velocity, and other properties. In the three systems, the number of ICM-102 molecules was not exactly the same. To facilitate comparison between the different systems, we divided the value of the heat release by the total number of ICM-102 molecules in the system to obtain the average heat release of each ICM-102 molecule. A comparison of the average heat release of each ICM-102 molecule in the pure ICM-102, ICM-102/H_2_O_2_, and ICM-102/HNO_3_ systems at different temperatures is shown in Fig. 2. Incorporation of the different guest molecules increased the heat release of the system. Moreover, the HNO_3_ guest molecule with stronger oxidation ability and higher O atom content than H_2_O_2_ more significantly increased the heat release of the host–guest system. A comparison of the heat release (at 3000 K) and detonation pressure/velocity values (cite from Ref.^2,3^) of the different host–guest systems are shown in Fig. 3. With increasing oxygen balance (OB) value of the system, both the heat release and detonation pressure values of the system greatly increased. Incorporation of H_2_O_2_ did not improve the detonation velocity. Except for the H_2_O_2_ guest molecule, the detonation velocity was positively correlated with the OB value. In the case that the OB values of pure ICM-102 and ICM-102/H_2_O_2_ are not much different, it is possible that the higher theoretical density of pure ICM-102 (~ 1.95 g·cm^−3^) leads to higher detonation velocity than ICM-102/H_2_O_2_.

### Mechanisms of the energy improvement of the host–guest explosives by the guest molecules

**Figure 4:**
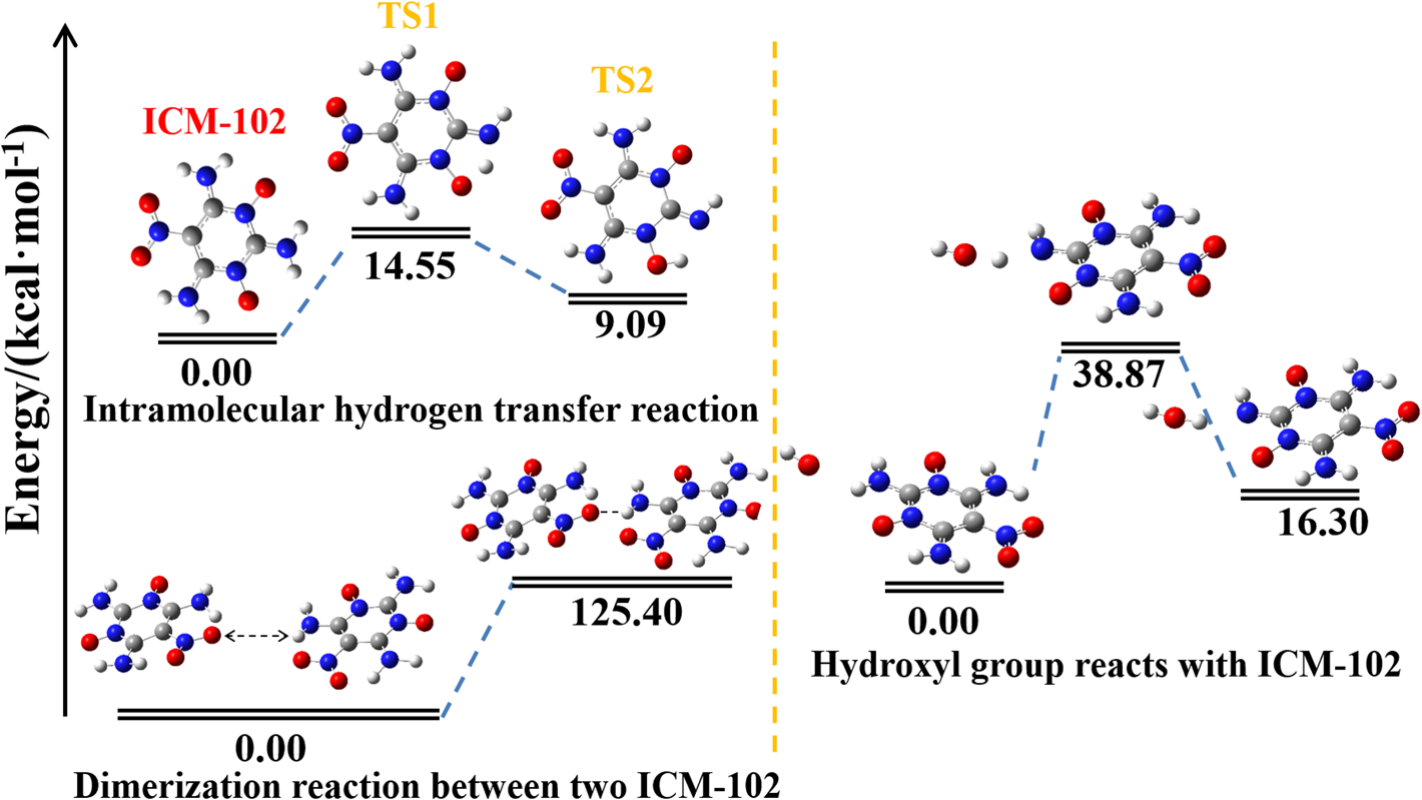
Energy barrier of the initial reaction pathway.

By processing the calculation results from LAMMPS by Python scripts, the primary reactions of the pure ICM-102, ICM-102/H_2_O_2_, and ICM-102/HNO_3_ systems were obtained, so as to determine the main initial reaction path in each system. In order to get correct frequency at which the reaction occurs, we selected the primary reactions where the difference between the forward and reverse reaction frequencies was greater than or equal to 5. The primary reactions of the pure ICM-102, ICM-102/H_2_O_2_, ICM-102/HNO_3_ systems, their lifetimes, and their occurrence frequencies in the first 10 ps at 3000 K are given in Table 2. To distinguish the same elements from the two components in the host–guest system, the elements in the guest molecules are placed in parentheses in Table 2. From the table, incorporation of the H_2_O_2_ and HNO_3_ guest molecules did not change the initial reaction path of ICM-102, which is consistent with the observed atomic trajectory. During the thermal decomposition reactions of the three systems, the intramolecular hydrogen transfer reaction of ICM-102 occurred first. The dimerization reaction between two ICM-102 molecules also occurred early. The chemical structures of some complex reactions were shown in Fig. S2. The energy barriers of the initial reaction pathway were calculated using Gaussian package with B3LYP functional and 6–311 + (d,p) basis sets, as shown in Fig. 4. In the host–guest systems, the decomposition of H_2_O_2_ and HNO_3_ both produced hydroxyl group. The H atoms of the amino groups of the ICM-102 molecules were attracted by the O atoms of hydroxyl group, which was the earliest formation path of water molecules. Subsequently, the main processes were reactions between small molecules.

**Table 2:** Primary reactions of the pure ICM-102, ICM-102/H_2_O_2_, and ICM-102/HNO_3_ systems at 3000 K.

System	Frequencies	Reaction time/ps	Primary reactions
Pure ICM-102	6	0.00–0.95	C_4_H_6_N_6_O_4_ + C_4_H_6_N_6_O_4_ → C_8_H_12_N_12_O_8_
	9	0.00–1.70	C_4_H_6_N_6_O_4_ → C_4_H_5_N_6_O_3_ + HO
	6	1.10–8.95	HO + HNO → NO + H_2_O
	6	1.25–9.25	H_3_NO → HO + NH_2_
	7	1.45–9.85	H_4_NO → HO + NH_3_
	6	2.55–8.90	CN_3_ → N_2_ + CN
	6	3.55–9.95	H_4_NO → H_2_O + NH_2_
ICM-102/H_2_O_2_	8	0.00–0.85	C_4_H_6_N_6_O_4_ + C_4_H_6_N_6_O_4_ → C_8_H_12_N_12_O_8_
	9	0.05–0.45	C_4_H_6_N_6_O_4_ + (HO) → C_4_H_5_N_6_O_4_ + H–(HO)
	6	0.05–0.95	C_4_H_5_N_6_O_3_ + (H_2_O_2_) → C_4_H_5_N_6_O_3_–(H_2_O_2_)
	10	0.05–1.15	C_4_H_6_N_6_O_4_–(HO) → C_4_H_5_N_6_O_4_ + H–(HO)
	9	0.05–3.75	C_4_H_6_N_6_O_4_ → C_4_H_5_N_6_O_3_ + HO
	6	1.95–9.65	H–(O) + H_2_O → H_3_O–(O)
	11	2.15–9.95	HNO_2_ → NO + HO
	6	2.55–8.00	HO + H_2_–(O) → H_2_O + H–(O)
	7	2.75–9.15	CN_3_ → N_2_ + CN
ICM-102/HNO_3_	6	0.00–0.75	C_4_H_6_N_6_O_4_ + C_4_H_6_N_6_O_4_ → C_8_H_12_N_12_O_8_
	5	0.00–0.45	C_4_H_6_N_6_O_4_ + (HNO_3_) → C_4_H_6_N_6_O_4_–(HO) + (NO_2_)
	22	0.00–8.95	(HNO_3_) → (NO_2_) + (HO)
	5	0.05–4.90	H–(HNO_3_) → (NO_2_) + H–(HO)
	7	0.10–0.70	C_4_H_6_N_6_O_4_ → C_4_H_5_N_6_O_3_ + HO
	7	0.30–9.90	H–(NO_2_) → (NO) + H–(O)
	5	0.45–7.15	(NO_2_) + HO → HO–(NO_2_)
	5	1.45–9.45	H_3_O–(O) → HO + H_2_–(O)
	7	1.70–9.90	HN_2_O → N_2_ + HO
	7	2.40–9.00	HNO_2_ → NO + HO

**Figure 5:**
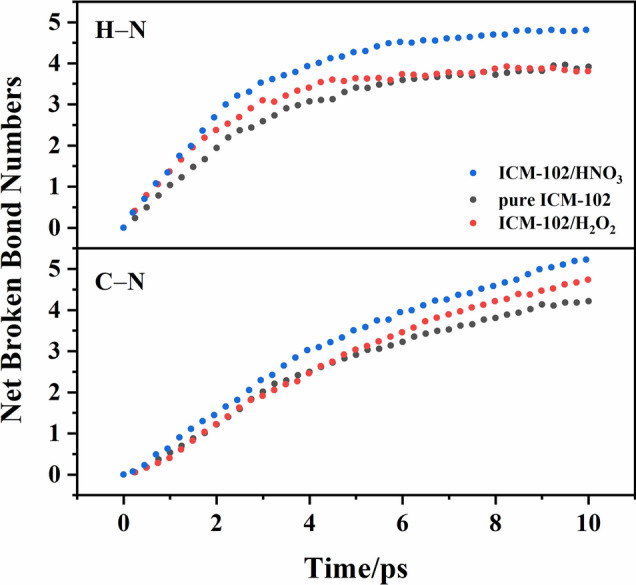
Changes of the cumulative sums of net broken C–N and H–N bonds with time in the pure ICM-102, ICM-102/H_2_O_2_, and ICM-102/HNO_3_ systems at 3000 K.

To further analyze the reaction mechanism of ICM-102 decomposition, we calculated the cumulative sums of the net broken bonds at different times, and then divided the cumulative sums by the total number of ICM-102 molecules in the initial system (144 for the pure ICM-102 and ICM-102/HNO_3_ systems, and 128 for the ICM-102/H_2_O_2_ system). Normalization was performed to facilitate direct comparison of the different systems. The changes of the cumulative sums of net broken C–N and H–N bonds with time in the pure ICM-102, ICM-102/H_2_O_2_, and ICM-102/HNO_3_ systems at 3000 K are shown in Fig. 5. C–N cleavage was the fastest and occurred the most in the ICM-102/HNO_3_ system, indicating that incorporation of the HNO_3_ guest molecule significantly accelerated thermal decomposition of the ICM-102 molecule. In the ICM-102/H_2_O_2_ system, the promoting effect of the guest H_2_O_2_ molecule on C–N bond cleavage was not obvious before about 4 ps, and the speed of C–N bond cleavage in the system was even slower than that in the pure ICM-102 system. After ~ 4 ps, the C–N bonds in the ICM-102/H_2_O_2_ system were rapidly broken, and the number of cleaved C–N bonds was also greater than that in the pure ICM-102 system. For the H–N bonds, the cleavage speed was also fastest in the ICM-102/HNO_3_ system. The promoting effect of the guest H_2_O_2_ molecule on H–N bond cleavage was mainly reflected before ~ 6 ps.

**Figure 6:**
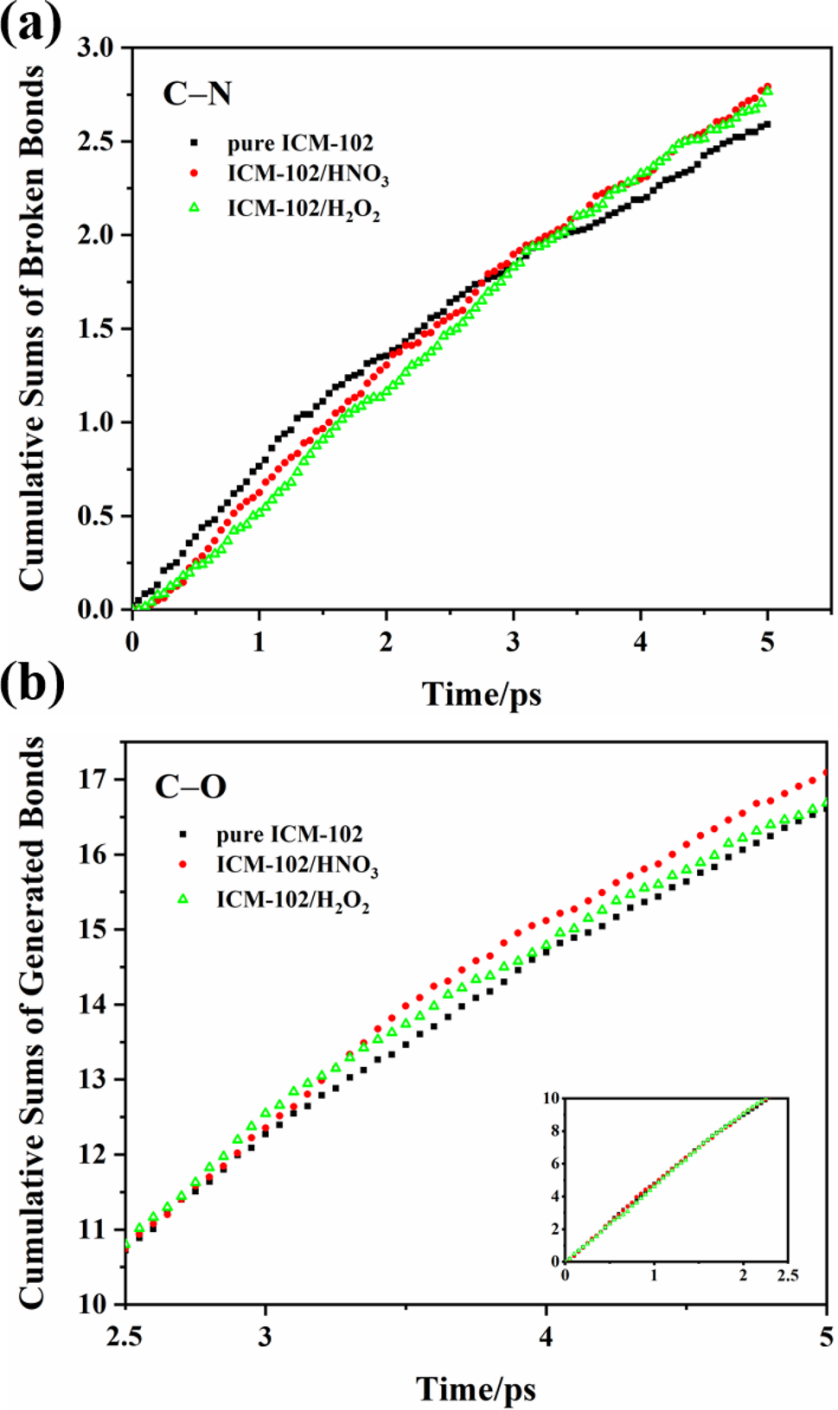
Changes of the cumulative sums of net generated/broken bonds with time in the pure ICM-102, ICM-102/H_2_O_2_, and ICM-102/HNO_3_ systems at 3000 K.

ICM-102 molecules contain three different types of C–N bonds: C–NO_2_, C–NH_2_, and C–N in the ring. To determine the influence of the guest molecules on the C–N in the ring, the C–N in the ring were counted separately (Fig. 6). Before about 3 ps, the numbers of broken C–N in the rings of the two host–guest systems were less than that in the pure ICM-102 system. After 3 ps, the numbers of broken C–N bonds in both the host–guest systems exceeded that in the pure ICM-102 system, indicating that the HNO_3_ and H_2_O_2_ guest molecules promoted C–N in the ring cleavage after about 3 ps. Generation of C–O bonds in the host–guest systems also exceeded that in the pure ICM-102 system after about 3 ps at 3000 K. This indicates that after 3 ps, the O atoms of the guest molecules mostly connected with C atoms of the ICM-102 ring, promoting C–N in the ring cleavage.

In summary, in terms of spatial geometries in host–guest systems, the hydrogen atom in the newly added guest molecules would form hydrogen bond with the oxygen atom located on the ring of ICM-102 molecules, which may hinder the intramolecular hydrogen transfer reaction to some extent. So that in the early stages, the amino group of ICM-102 remains intact. After a period of time, both HNO_3_ and H_2_O_2_ molecules would decompose to produce free hydroxyl group. The hydroxyl group would promote H–N bond cleavage of the amino groups of the ICM-102 molecules, which is the earliest effect of guest molecules on decomposition of ICM-102.

### Effect of the different guest molecules on the reaction kinetic parameters

**Figure 7:**
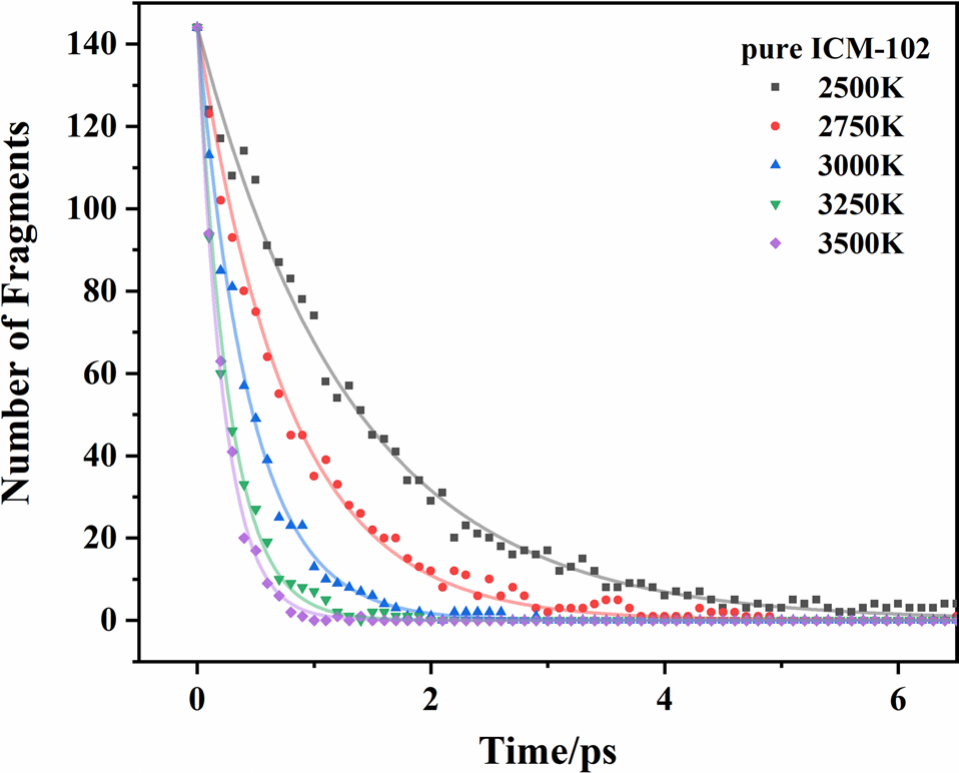
Change of the number of ICM-102 molecules with time in the pure ICM-102 system at different temperatures. The lines are the fitted curves.

**Figure 8:**
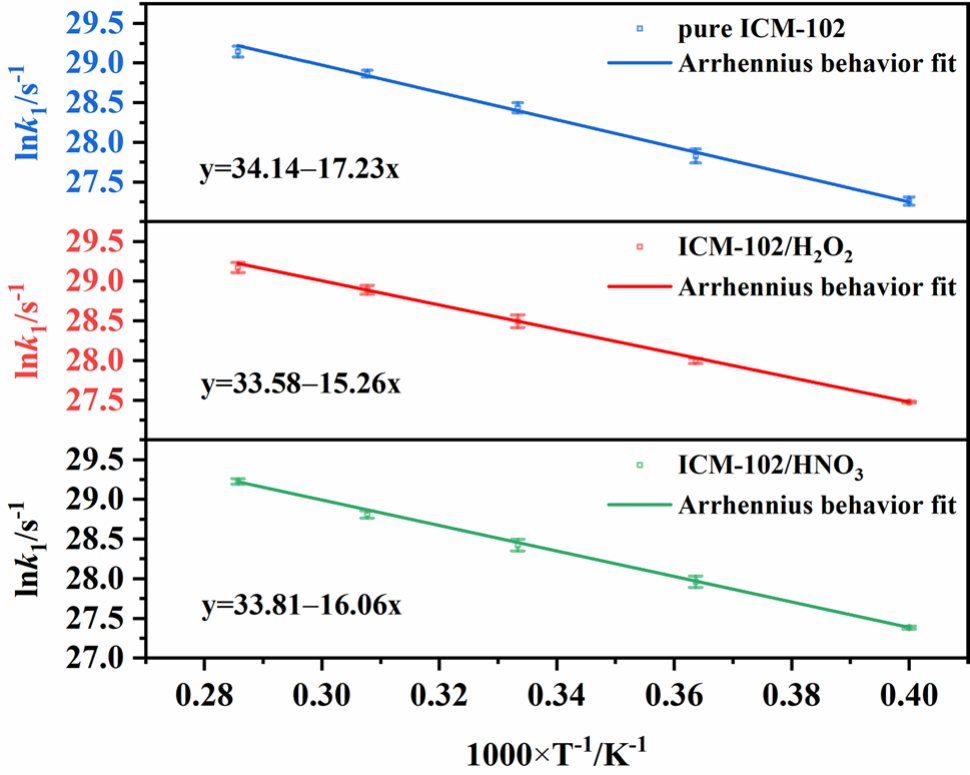
Logarithm of *k*_1_ plotted against the inverse temperature (1/*T*) at 2500, 2750, 3000, 3250, and 3500 K.

The change of the number of ICM-102 molecules with time in the pure ICM-102 system at 3000 K is shown in Fig. 7. The fitted curves are basically consistent with the curves of the molecular numbers, indicating that the decrease of the number of ICM-102 molecules conforms to a first-order decay exponential function. In the ICM-102/HNO_3_ and ICM-102/H_2_O_2_ host–guest systems, the changes of the number of ICM-102 molecules with time are shown in Figs. S3 and S4. The logarithm of *k*_1_ plotted against the inverse temperature (1/*T*) at 2500, 2750, 3000, 3250, and 3500 K for the three systems is shown in Fig. 8. The initial thermal decomposition processes agree with the Arrhenius law. The fitted initial decomposition activation energy (*E*_a1_) values of the pure ICM-102, ICM-102/H_2_O_2_, and ICM-102/HNO_3_ systems are 143.25, 126.87, and 133.52 kJ mol^−1^, respectively. Incorporation of guest molecules increased the decomposition rate of ICM-102 in the initial endothermic stage.

**Figure 9:**
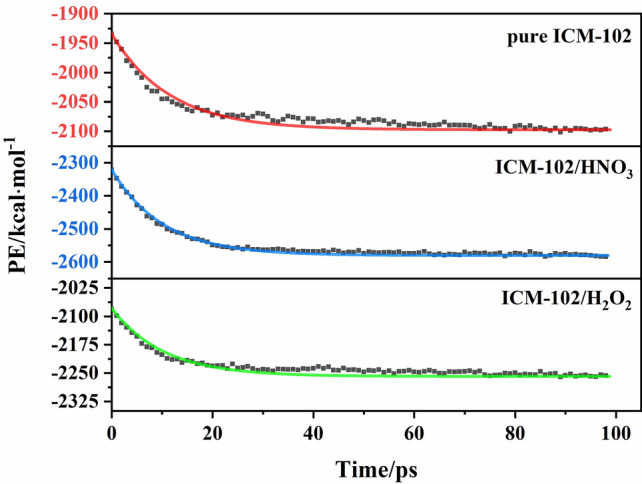
Evolution of the PE with time for the pure ICM-102, ICM-102/H_2_O_2_, ICM-102/HNO_3_ systems at 3000 K. The lines are the fitted curves.

**Figure 10:**
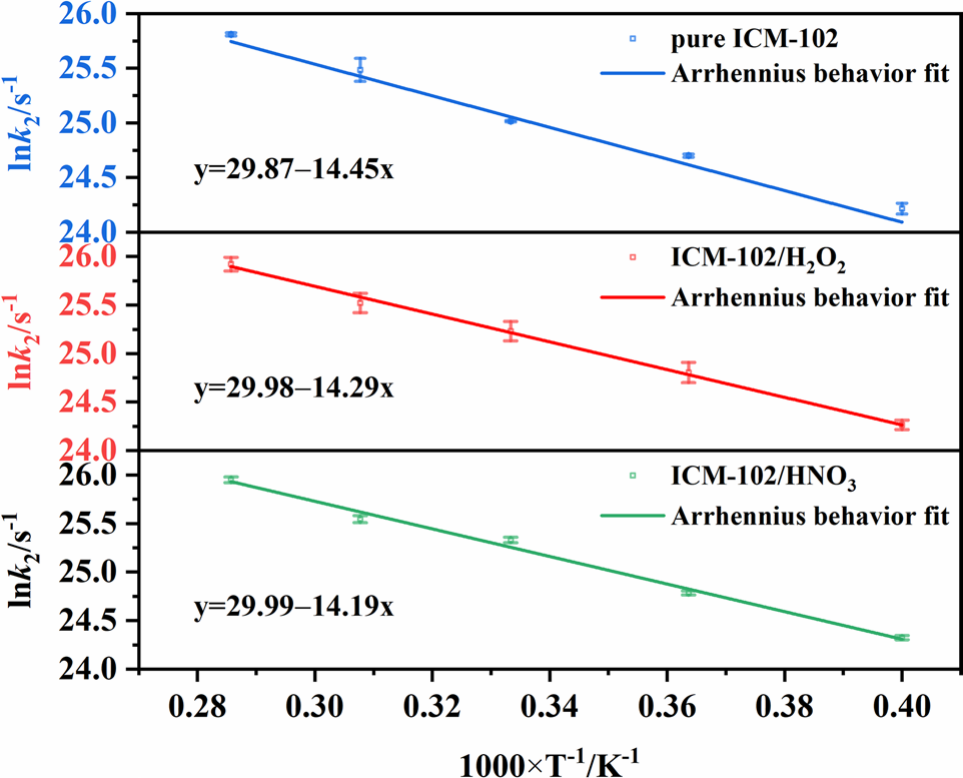
Logarithm of *k*_2_ plotted against the inverse temperature (1/*T*) at 2500, 2750, 3000, 3250, and 3500 K.

According to Eq. (2), the PE curves were fitted to obtain the exothermic stage reaction rates (*k*_2_) at different temperatures. The evolution of the PE with time and the fitted curves for the pure ICM-102, ICM-102/H_2_O_2_, ICM-102/HNO_3_ systems at 3000 K are shown in Fig. 9. The fitted curves are basically consistent with the PE curves, indicating that the intermediate decomposition conforms to a first-order reaction rate equation. The logarithm of *k*_2_ plotted against the inverse temperature (1/*T*) at 2500, 2750, 3000, 3250, and 3500 K for the three systems is shown in Fig. 10. The intermediate decomposition processes agree with the Arrhenius law. The fitted intermediate decomposition activation energy (*E*_a2_) values of the pure ICM-102, ICM-102/H_2_O_2_, and ICM-102/HNO_3_ systems are 120.14, 118.81, and 117.98 kJ mol^−1^, respectively. Incorporation of the guest molecules greatly improved the reaction rate of the system in the exothermic stage, and HNO_3_ had a more obvious promoting effect on the energy release of the system.

**Figure 11:**
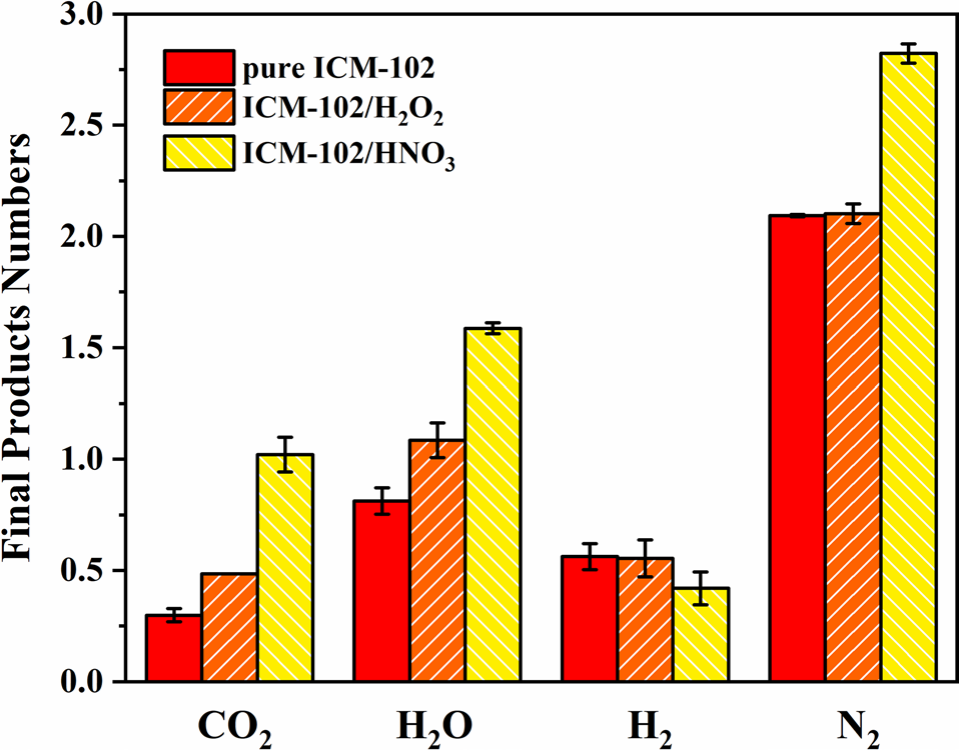
Comparison of the numbers of CO_2_, H_2_O, H_2_, and N_2_ molecules in the pure ICM-102, ICM-102/H_2_O_2_ and ICM-102/HNO_3_ systems at 3000 K.

By analyzing the calculated products, we found that the main final molecular products were CO_2_, H_2_O, H_2_, and N_2_. A comparison of the numbers of CO_2_, H_2_O, H_2_, and N_2_ molecules in the pure ICM-102, ICM-102/H_2_O_2_, and ICM-102/HNO_3_ systems at 3000 K is shown in Fig. 11. The order of the CO_2_ and H_2_O molecule numbers in the three systems was ICM-102/HNO_3_ > ICM-102/H_2_O_2_ > pure ICM-102. The guest oxidizing small molecules had a significant promoting effect on formation of CO_2_ and H_2_O. In addition, the promoting effect was greater for higher oxygen content of the guest molecule. However, the number of H_2_ molecules in the pure ICM-102 system was slightly greater than those in the two host–guest systems. This is because incorporation of guest molecules increases the oxygen balance of the system, leading to a decrease in the amount of incomplete reaction products (such as hydrogen gas) in the system. The number of N_2_ molecules in the ICM-102/HNO_3_ system was significantly higher than those in the other two systems. This is because the HNO_3_ guest molecule contains nitrogen, and more N_2_ is generated during the reaction.

**Figure 12:**
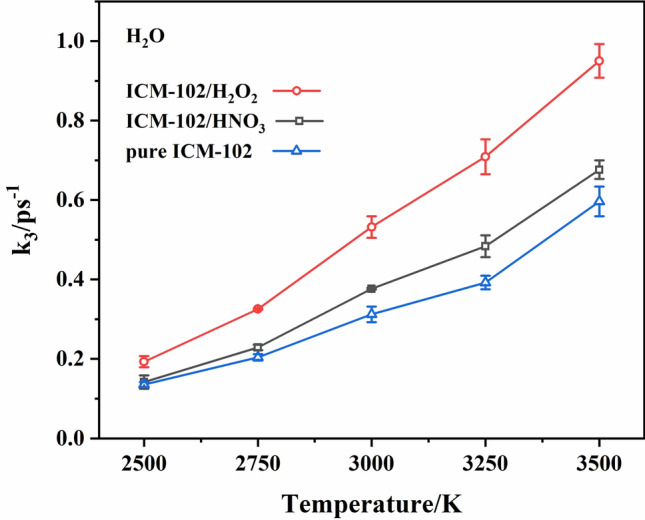
Comparison of the H_2_O formation rates in the pure ICM-102, ICM-102/H_2_O_2_ and ICM-102/HNO_3_ systems at different temperatures.

H_2_ was generated in a small amount compared with CO_2_, H_2_O, and N_2_. The H_2_ formation rates of the three systems were similar, so fitting analysis of the H_2_ formation rate was not performed. A comparison of the H_2_O formation rates in the pure ICM-102, ICM-102/H_2_O_2_, and ICM-102/HNO_3_ systems at different temperatures is shown in Fig. 12. At different temperatures, the order of the H_2_O formation rates in the three systems was ICM-102/H_2_O_2_ > ICM-102/HNO_3_ > pure ICM-102. Moreover, the H_2_O_2_ guest molecule accelerated H_2_O formation much more than the HNO_3_ guest molecule.

**Figure 13:**
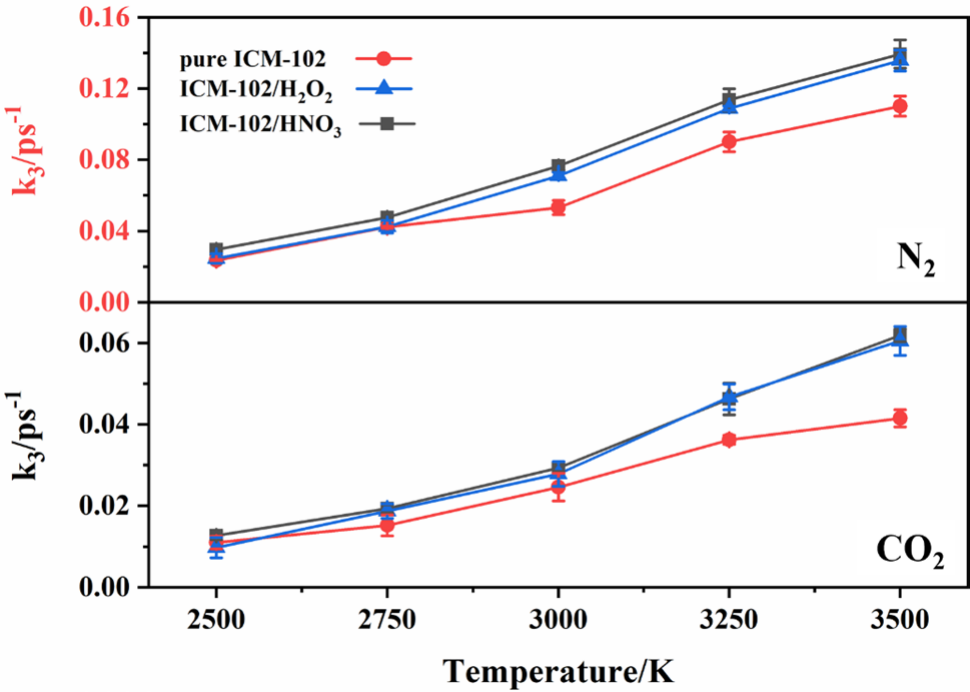
Comparison of the N_2_ and CO_2_ formation rates in the pure ICM-102, ICM-102/H_2_O_2_ and ICM-102/HNO_3_ systems at different temperatures.

A comparison of the N_2_ and CO_2_ formation rates in the pure ICM-102, ICM-102/H_2_O_2_, and ICM-102/HNO_3_ systems at different temperatures is shown in Fig. 13. The promoting effect of the HNO_3_ guest molecule on N_2_ formation was obvious at all of the temperatures. However, H_2_O_2_ had little effect on the formation rate of N_2_ at low temperature. At relatively high temperatures (3000 K and above), the promoting effect of H_2_O_2_ on N_2_ formation was obvious. For CO_2_ molecules, the two host–guest systems showed an obvious promoting effect on its formation at different temperatures, and the promoting effect was greater for higher temperature.

### Effect of guest molecules on the thermal stabilities of the host–guest systems

**Figure 14:**
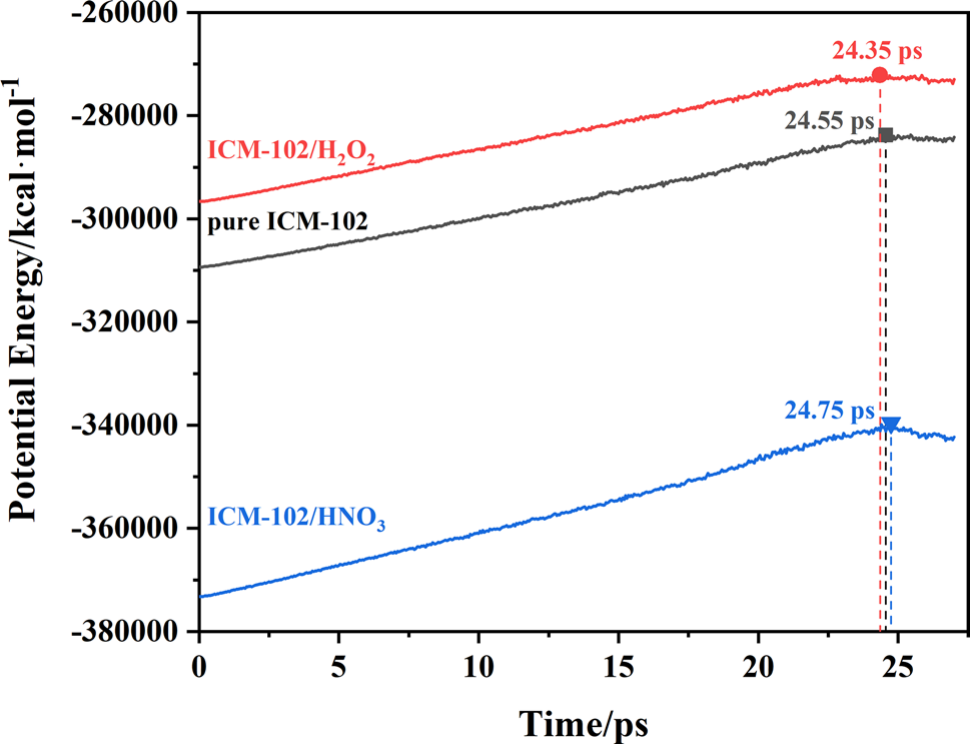
Evolution of the PE with time in the pure ICM-102, ICM-102/H_2_O_2_, and ICM-102/HNO_3_ systems at 100 K ps^−1^ heating rate.

The thermal stability of different systems could sort through the time sequence when the system starts to release heat at a same heating rate. The time of system start to release heat corresponds to the decrease of the system potential energy. The pure ICM-102, ICM-102/H_2_O_2_, and ICM-102/HNO_3_ systems were heated from 300 to 3000 K at different heating rates. The evolution of the PE with time in the pure ICM-102, ICM-102/H_2_O_2_, and ICM-102/HNO_3_ systems at heating rates of 100 and 50 K ps^−1^ is shown in Figs. 14 and S5, respectively. The temperature of the system continuously increased under the programmed heating, and the PE value also constantly increased. The PE values of the three systems all increased to a maximum. The PE then started to decrease, which means that the system began to decompose and release heat. By comparing the results under programmed heating at different heating rates, we found that the system started to decompose at lower temperature for lower heating rate. Incorporation of guest molecules did not significantly change the time when the system began to decompose because the decomposition times in the different systems were almost the same. Therefore, we believe that the different guest molecules had little effect on the thermal stability of the system.

## Conclusions

ReaxFF-lg MD simulations have been performed to investigate the mechanism of the improvement of the energy of host–guest explosives by guest molecules under different constant high temperatures and different heating rate. The effects of different guest molecules on the initial reaction mechanisms and reaction stages of the systems were analyzed in detail.

Incorporation of guest molecules significantly increased the energy levels of the host–guest systems. However, the initial reaction paths of the ICM-102 molecule in the different systems were similar. The guest molecules did not initially participate in the host molecule reaction. After a period of time, the H_2_O_2_ and HNO_3_ guest molecules promoted cleavage of the C–N bond of the ICM-102 ring. Destruction of the ICM-102 ring structure was more obviously accelerated for the guest molecule with stronger oxidation ability and higher oxygen content. At different high temperatures, the intramolecular hydrogen transfer reaction and dimerization reaction between ICM-102 molecules occurred first. Combined with analysis of kinetic parameters, the guest molecules accelerated the initial endothermic reaction of ICM-102, but they played a more important role in the intermediate exothermic reaction stage: incorporation of guest molecules (HNO_3_ and H_2_O_2_) greatly improved the heat release in the system and the chemical reaction rate in the exothermic reaction stage. The promoting effect of more oxidizing HNO_3_ was greater than that of H_2_O_2_. Although the number and formation rate of the main final products N_2_, H_2_O, and CO_2_ improved, the guest molecules HNO_3_ and H_2_O_2_ had a more obvious promoting effect on formation of N_2_ and H_2_O, respectively. Incorporation of guest molecules had little effect on the thermal stabilities of the systems.

The rule of the influence of different guest molecules on the thermal reaction could guide selection of guest molecules in synthesis of host–guest materials.

## Supplementary Information

Supplementary Information.
