# Supplementary material for: Mechanism of the improvement of the energy of host–guest explosives by incorporation of small guest molecules: HNO3 and H2O2 promoted C–N bond cleavage of the ring of ICM-102
Source: Sci Rep. 2021 May 18;11:10559. doi: 10.1038/s41598-021-89939-1 (PMC8131615; doi:10.1038/s41598-021-89939-1)
Supplement: Supplementary file 1 — Supplementary Information. [file 41598_2021_89939_MOESM1_ESM.docx]

**Mechanism of the Improvement of the Energy of Host–Guest Explosives by Incorporation of Small Guest Molecules: HNO_3_ and H_2_O_2_ Promoted C–N Bond Cleavage of the Ring of ICM-102**

*Yiwen Xiao, Lang Chen,* Kun Yang, Deshen Geng, Jianying Lu, Junying Wu*

State Key Laboratory of Explosion Science and Technology, Beijing Institute of Technology, Beijing 100081, China

Corresponding Author: Lang Chen, Tel: +86 10 68914711.

E-mail: chenlang@bit.edu.cn


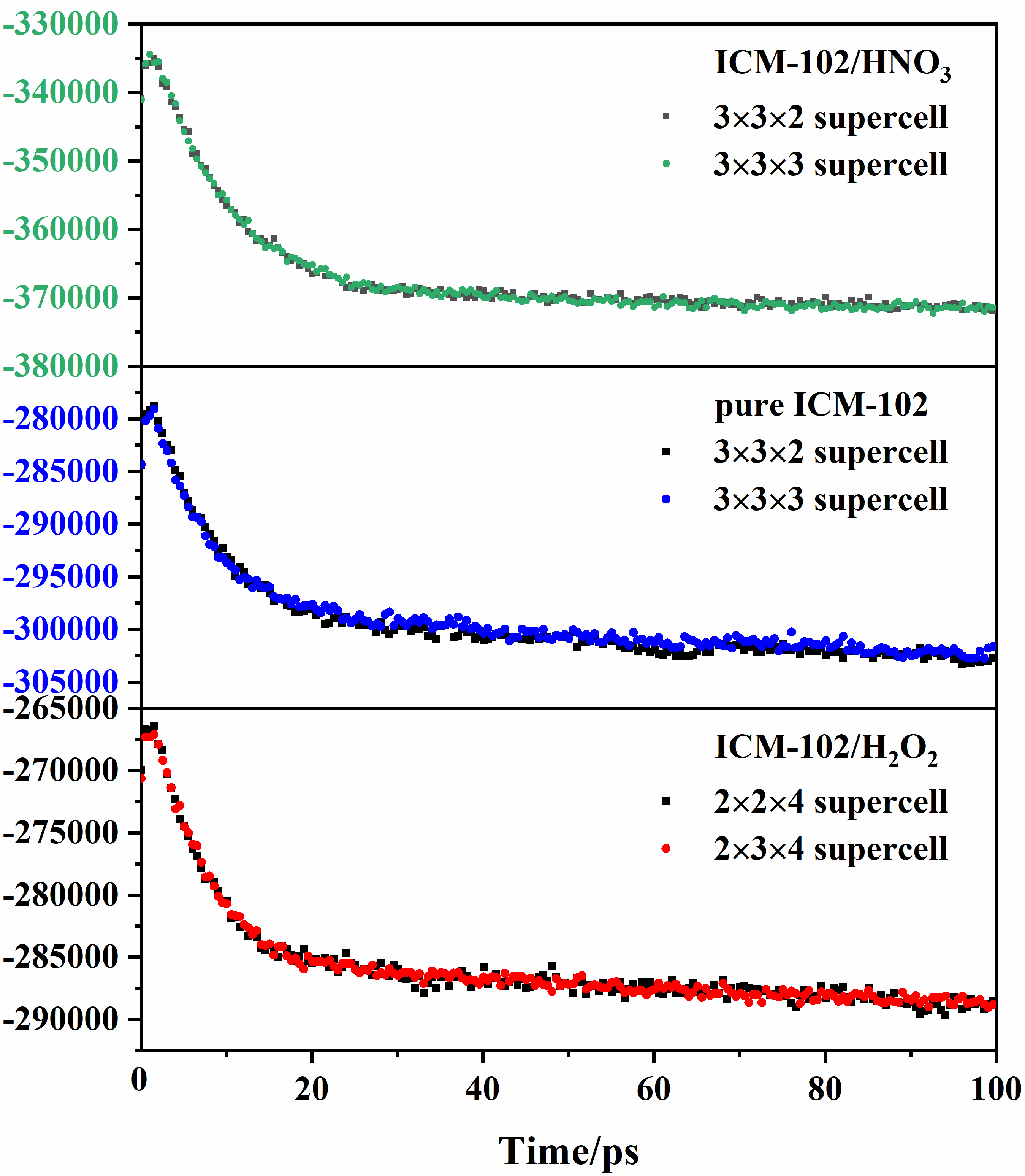


Figure S1. The comparison of the evolution of potential energy of the large and small systems with time at 3000 K.


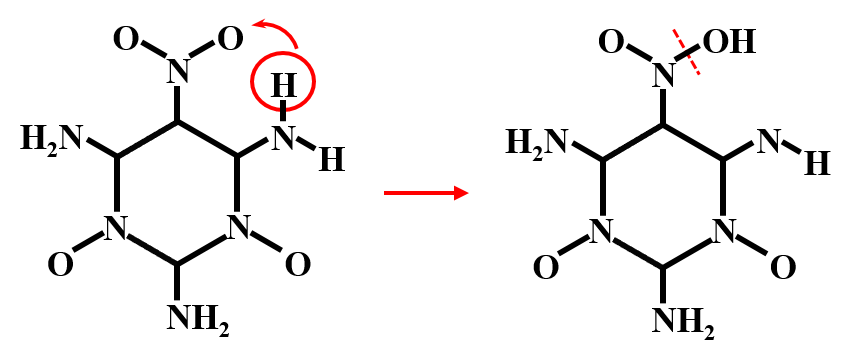


**(a) C_4_H_6_N_6_O_4_ → C_4_H_5_N_6_O_3_ + HO**


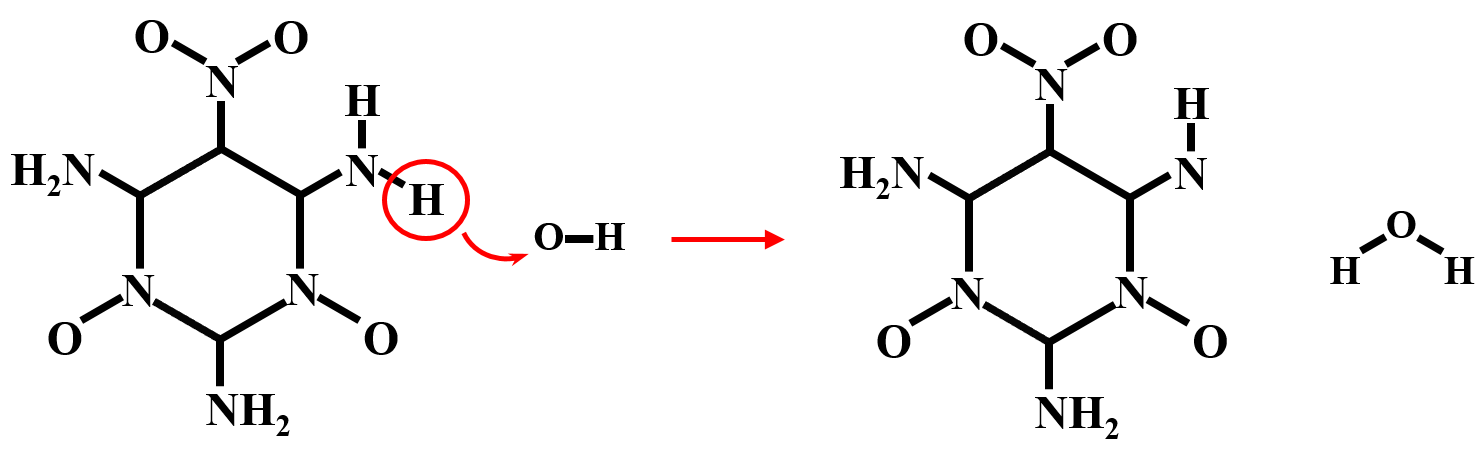


**(b) C_4_H_6_N_6_O_4_ + (HO) → C_4_H_5_N_6_O_4_ + H–(HO)**

Figure S2. Chemical structure of reactions.


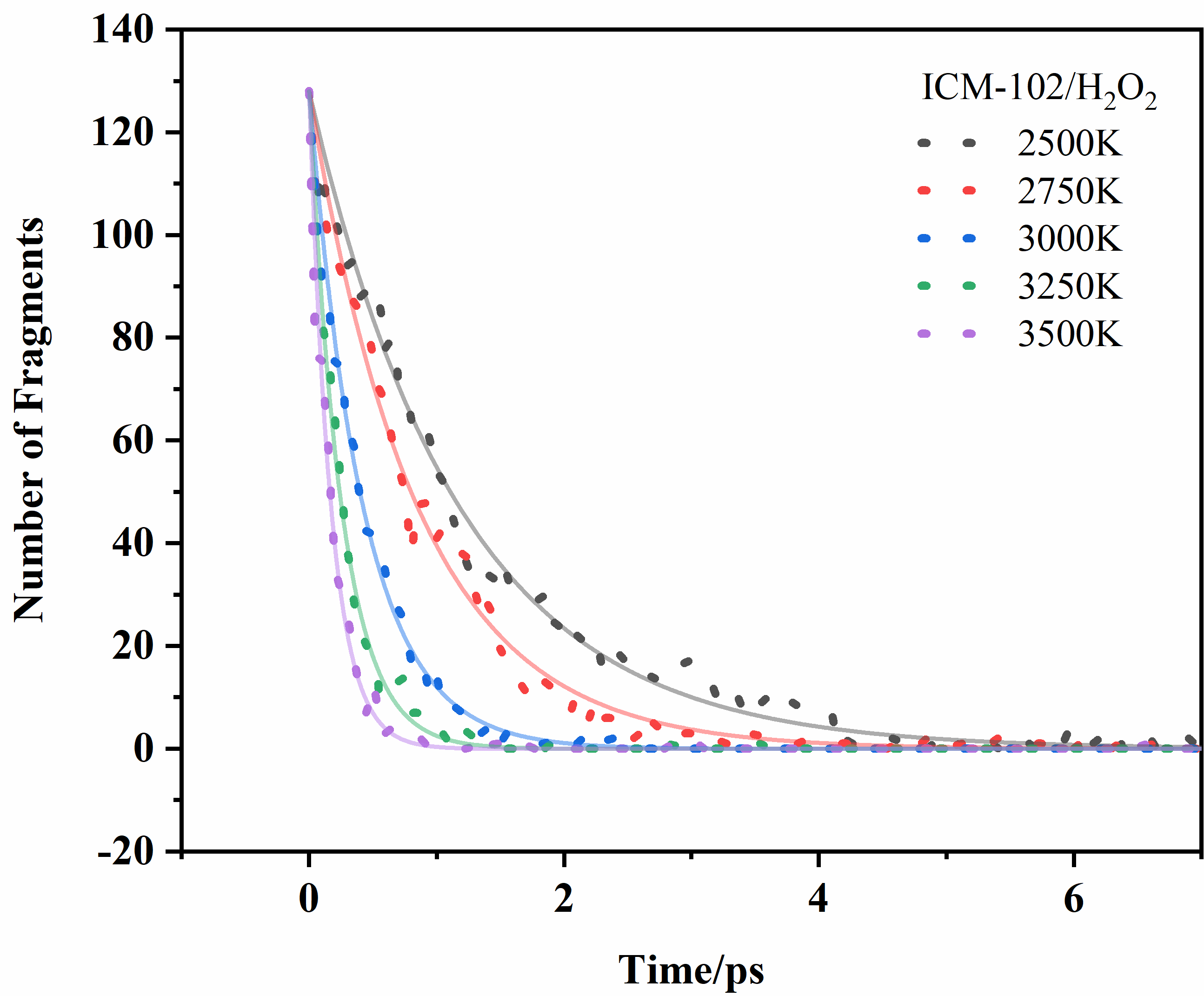


Figure S3. Change of the number of ICM-102 molecules with time in the ICM-102/H_2_O_2_, system at different temperatures. The lines are the fitted curves.


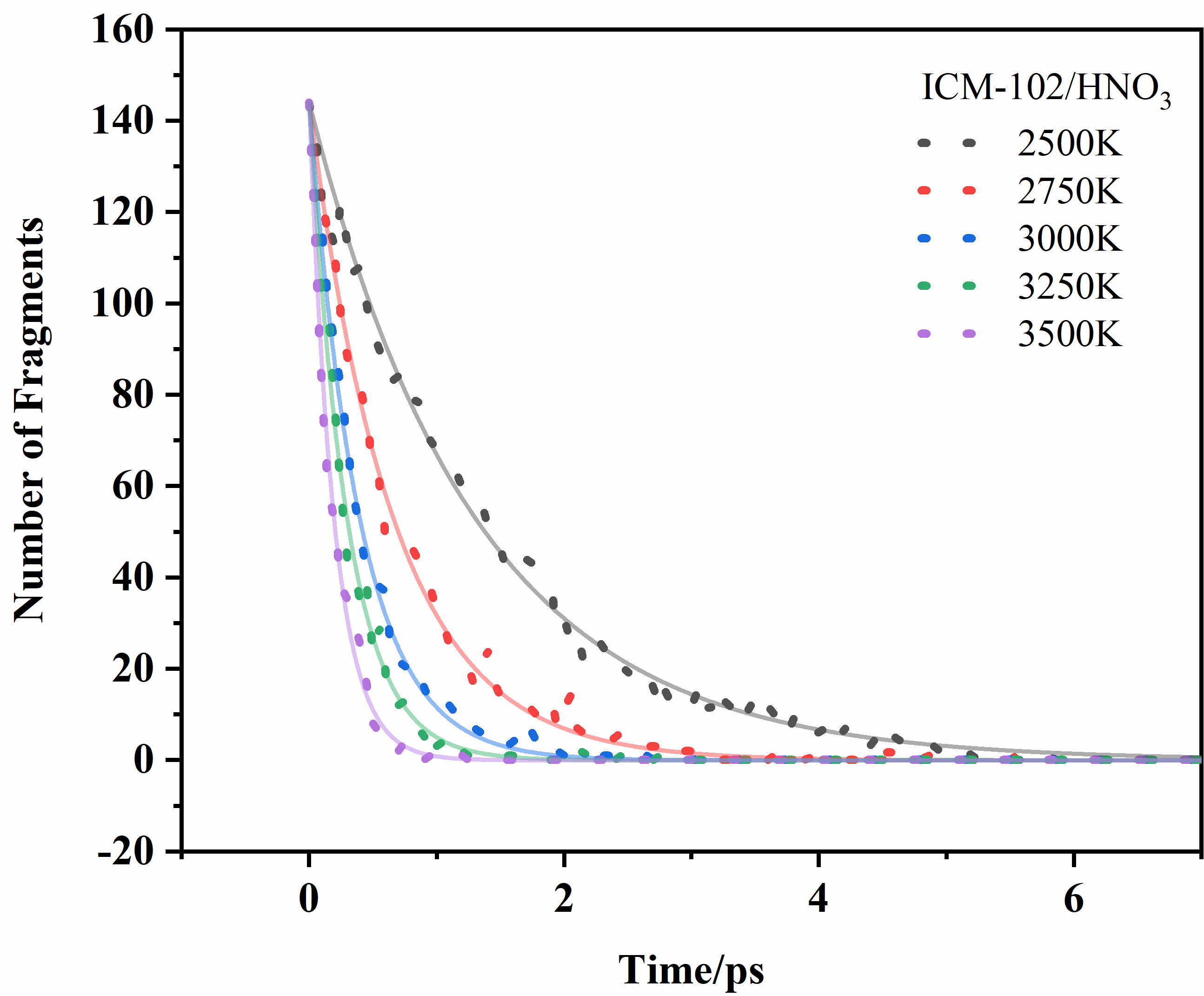


Figure S4. Change of the number of ICM-102 molecules with time in the ICM-102/HNO_3_ system at different temperatures. The lines are the fitted curves. The fitted curves are basically consistent with the curves of the molecular numbers.





Figure S5. Evolution of the PE with time in the pure ICM-102, ICM-102/H_2_O_2_, and ICM-102/HNO_3_ systems at 50 K/ps heating rate. By comparing the results under programmed heating at different heating rates, we found that the system started to decompose at lower temperature for lower heating rate.
